# Response to: “letter to the editor regarding the article “the global distribution of acute unintentional pesticide poisoning: estimations based on a systematic review”” by Dunn et al. 2021 in BMC public health

**DOI:** 10.1186/s12889-021-11941-z

**Published:** 2021-10-27

**Authors:** Wolfgang Boedeker, Meriel Watts, Peter Clausing, Emily Marquez

**Affiliations:** 1 Pesticide Action Network (PAN) Germany, Nernstweg 32, 22765 Hamburg, Germany; 2 Pesticide Action Network (PAN) Asia Pacific, P.O. Box 1170, 10850 Penang, Malaysia; 3 Pesticide Action Network (PAN) North America, 2029 University Ave., Suite 200, Berkeley, CA 94704 USA

**Keywords:** Pesticide, Insecticide, Herbicide, Poisoning, Mortality, Morbidity, Occupational, Farmer, Farmworker, Agriculture

## Abstract

In a correspondence to BMC Public Health, Dunn et al. (Dunn SE, Reed J and Neumann C. BMC Public Health (n.d)) respond to our review on the occurrence of unintentional, acute pesticide poisoning (UAPP). Based on a systematic review and further data sources we estimated that about 385 million cases of UAPP occur annually world-wide including around 11,000 fatalities (Boedeker W. et al. BMC Public Health:1875, 2020).

## Main text

Dunn et al. [1] question the results of our study [2] and elaborate on two examples for a suggested general overestimation. We reply to these examples first and follow with response to the more general comments of the critics.

Dunn et al. state: “We are uncertain as to how they arrived at these figures though a substantial proportion of the numbers appear to have been extracted from inflated US data”. This is not correct. Our estimation of UAPP made no use of this data source because we restricted non-fatal UAPP to the occupational/farming population, which is not specified in the reports of the US Poison Control Centers. However, if we were interested in the general population in this respect, we would use the US Poison Control Center reports as a welcomed and valid input. This is because Dunn et al. err in two more aspects. First, the reports do specify intentional as well as unintentional UAPP (see Table 22A Mowry et al. [3]). Second, a restriction to incidents with documented symptom severity– as thought necessary by the critics – would lead to underreporting, as the follow-up of the medical outcomes could be done by the Poison Control Centre in less than 50% of the cases.

Dunn et al. use the data coverage of Western Africa as an example of an inflated extrapolation. They suggest that overestimation of fatal UAPP follows from having data from just 0.15% of its population. Unfortunately, they choose not to mention that our estimate of fatal UAPP in Western Africa is zero. Data were available for Cabo Verde only, which reported no fatalities of UAPP to WHO (see Suppl S3b of our paper). Furthermore, Dunn et al. do not seem to have understood our estimation method. In contrast to their apprehension “When no data were available for a particular country, the authors extrapolated using UAPP frequencies from other geographies”, we did not extrapolate national figures from data of other countries. If there were no data available for a country, no national figures were derived. We detailed our estimation method explicitly. Along with the data presented in our paper and the supplements, the estimates can easily be reproduced using a pocket calculator.

When it comes to further points raised by Dunn et al., we refer to our article where we have elaborated at length on the issues of case definition, validity of data sources, representativity, and their possible influence on our estimations. We also acknowledged reasons for over- and underreporting and tried to assess bias by sensitivity analyses. Finally, we think that our figures have been derived by valid methods and procedures and fill a research gap that has been left open for 30 years. The estimates are the best we could arrive at on the basis of available data but should still not be reported with too many decimals.

Our article is on the estimation of the annual world-wide UAPP, with a focus on the farming population. Still, Dunn et al. feel that addressing the benefits of pesticides “is important for a complete evaluation”, a weird public health perspective often taken by the pesticide industry to obscure issues. We fail to see how millions of UAPP could be balanced by millions of malaria cases or malnourished people. However, even in these areas, there is scientific consensus that pesticides are not just a simple cure but part of the problem. For example, the prevention of malaria relies on a complex understanding of the vector ecology, local needs, and environmental conditions but replacement of this approach leads to a simplified dependency on insecticides and severe new problems, e.g. by insecticide-resistant vectors [4].

Also, it is common knowledge by now, that the cause of malnutrition and hunger is not a lack of pesticides but of availability and not production of food. Dunn et al. might get the idea from the Special Rapporteur of the UN General Assembly who critiqued “… the expansion of an international economic regime that promotes the unequal distribution of resources, the exploitation of agricultural workers, a rise in monocultural production and a lessening of diversity in food systems in times of climate emergency. … Investment should be diversified and reconciled with more responsible and sustainable food system methodologies, such as agroecology, as well as traditional knowledge. That requires a well-conceived shift away from industrial agriculture, which constitutes the main driver of the climate emergency, coupled with the promotion of transformative, resilient and sustainable practices. Agroecology avoids the use of dangerous biochemicals and pesticides; supports the local food movement; protects smallholder farmers, including women, and small fisheries; respects human rights; enhances food democracy, traditional knowledge and culture; maintains environmental sustainability; and helps to facilitate a healthy diet” [5].

Lastly, we welcome the ongoing evaluation of new and existing pesticides against the FAO/WHO Code on Conduct on Pesticide Management, especially Article 3.6 which states that “Pesticides whose handling and application require the use of personal protective equipment that is uncomfortable, expensive or not readily available should be avoided, especially in the case of small-scale users and farm workers in hot climates” [6]. Implementation of this article would likely result in a dramatic reduction in UAPP.

## Data Availability

Not applicable.

## Abbreviations

FAO: Food and Agricultural Organization of the United Nations
PAN: Pesticide Action Network
UAPP: unintentional acute pesticide poisoning
UN: United Nations
WHO: World Health Organization
